# Cumulative evidence for the association between maternal hypertension and cleft lip and palate in offspring: a systematic review and meta-analysis

**DOI:** 10.3389/froh.2026.1725513

**Published:** 2026-03-13

**Authors:** Xinping Cui, Bin Song, Qian Zhou, Siyu Yang, Yan Zeng

**Affiliations:** 1 North Sichuan Medical College, Nanchong, China; 2 Department of Nephrology, People’s Hospital of Deyang City, Deyang, China; 3 Southwest Medical University, Luzhou, China; 4 Chengdu University of Traditional Chinese Medicine, Chengdu, China; 5 Department of Pediatrics, People’s Hospital of Deyang City, Deyang, China

**Keywords:** cleft lip and palate, hypertension, meta-analysis, offspring, risk factors

## Abstract

**Objective:**

Maternal hypertension has serious maternal and neonatal consequences. Evidence regarding the relationship between maternal hypertension and cleft lip and palate in offspring is limited and inconsistent. Therefore, we conducted a meta-analysis to evaluate the relationship between maternal hypertension and the risk of cleft lip and palate in offspring.

**Methods:**

We systematically searched the following English electronic databases to identify relevant observational studies for our analysis: PubMed, Cochrane Library, Embase and Web of Science, supplemented by a manual reference researches. Study screening, data extraction, and risk-of-bias assessments were conducted independently by two researchers. A meta-analysis was performed using Stata 18.0 and Revman 5.3. Heterogeneity (I^2^ statistics) and sensitivity analyses were carried out to ensure analytical rigor and robustness.

**Results:**

The meta-analysis ultimately included 12 eligible studies, comprising 4,407,254 participants. Under the random-effects model, the analysis indicated an association between maternal hypertension and cleft lip and palate in offspring [OR = 1.44, 95% CI = 1.26, 1.64, *p* = 0.01]. Both maternal gestational hypertension and preeclampsia were associated with an increased risk of cleft lip and palate in offspring (gestational hypertension: OR = 1.36, 95% CI = 1.06, 1.74, *p* = 0.01, preeclampsia: OR = 2.10, 95% CI = 1.11, 3.96, *p* = 0.02). Sensitivity analyses supported the robustness of our results.

**Conclusion:**

With our meta-analysis, we provide evidence for an association between maternal hypertension (particularly gestational hypertension and preeclampsia) and CLP in children. Given that residual confounding cannot be entirely excluded, caution is warranted in interpreting the conclusions of this study. More high-quality studies are needed to establish whether the association with maternal hypertension is causal.

**Systematic Review Registration:**

PROSPERO CRD420251152806.

## Introduction

The high visibility and prevalence of congenital cleft lip and palate (CLP) at birth have made it one of the most extensively studied congenital abnormalities (1). Cleft lip with or without cleft palate occurs due to abnormal embryonic development of the lip and palate (2). CLP can be classified into three categories: isolated cleft lip (CL), isolated cleft palate (CP), and cleft lip with cleft palate. The global prevalence rate is 0.1244%, which equates to 1 to 1.3 cases per 1,000 live births (3). CLP can cause abnormal facial appearance, speech and feeding difficulties, hearing problems, abnormal tooth development, and otitis media. CLP may result in psychological problems such as low self-esteem, anxiety, and depression, thereby impairing social skills and overall mental health. These complications impose a heavy burden on individuals and society (4).

CLP is caused by multiple factors, with genetic and environmental factors being the most significant. The incidence of maternal hypertension is continuously rising, constituting a growing public health concern. It is recognized as a significant risk factor for CLP. Maternal hypertension, encompassing pre-eclampsia, is a widely acknowledged and severe gestational complication. It precipitates uteroplacental insufficiency, fetal hypoxia, and oxidative stress—all of which are postulated as pathological mechanisms capable of compromising the trajectory of normal embryonic craniofacial morphogenesis. Recent studies have suggested a possible link between maternal hypertension and CLP risk in offspring (5, 6). However, other studies have reported inconsistent results (7, 8). The existing observational studies have shown significant heterogeneity in their conclusions. This may be due to confounding factors in observational studies. Thus, the correlation between maternal hypertension and CLP risk in offspring remains controversial. Therefore, we conducted a meta-analysis to synthesize existing evidence and evaluate the association between maternal hypertension and CLP in offspring. It seeks to provide a pooled effect estimate and quantify the associated confidence intervals, thereby addressing the conflicting findings and uncertainty stemming from individual studies.

## Rationale and scientific gap

Cleft lip and palate (CLP) is a multifactorial congenital anomaly driven by both genetic susceptibility and environmental exposures. Several well-established maternal risk factors—including diabetes mellitus, cigarette smoking, obesity, folate deficiency, and a positive family history—have been extensively characterized in the literature. By contrast, maternal hypertension has received comparatively limited systematic investigation, despite its high and rising global prevalence and distinct pathophysiological profile in pregnancy. To our knowledge, no prior meta-analysis has quantitatively evaluated maternal hypertension as an independent exposure while distinguishing between hypertensive phenotypes and rigorously addressing confounding via subgroup and meta-regression analyses. Previous reviews have either focused on antihypertensive medication use or grouped hypertension with broader metabolic conditions, thereby masking the independent contribution of hypertensive disorders themselves. This analytical gap constrains causal interpretation and risk stratification in prenatal care, which the present study aims to address.

Maternal hypertension was selected *a priori* as the primary exposure because it constitutes a clinically defined vascular-placental disorder with distinct pathophysiology—uteroplacental malperfusion, endothelial dysfunction, and hypoxia-related stress—that differs from behavioral exposures (e.g., smoking) and metabolic disorders (e.g., diabetes). Furthermore, hypertensive disorders of pregnancy are routinely screened for in prenatal care, supporting potential translation to clinical risk stratification if an independent association is confirmed. Accordingly, this meta-analysis was designed to evaluate hypertension as a standalone exposure by prioritizing multivariable-adjusted effect estimates and stratifying analyses by hypertensive phenotype (gestational hypertension, preeclampsia, and pre-pregnancy/chronic hypertension), thereby minimizing exposure misclassification and clarifying phenotype-specific risk patterns.

## Research methods

### Registration

Our meta-analysis followed the MOOSE guidelines (21). This meta-analysis was registered on PROSPERO, an international systematic review registry (CRD420251152806). The key elements prespecified in the protocol registered prior to study initiation were as follows: (1) Primary outcome: pooled odds ratios (ORs) estimating the association between maternal hypertension exposure and the risk of cleft lip and palate (CLP) in offspring. (2) Prespecified subgroup analyses: analyses stratified by type of maternal hypertension, study quality, study design, and the extent of adjustment for confounding factors. (3) Prespecified sensitivity analyses: analyses planned to assess the robustness of the findings by excluding studies of low methodological quality and by comparing results derived from random-effects and fixed-effects models.

### Literature search strategy

A systematic search was performed in four electronic databases: PubMed, Cochrane Library, Embase, and Web of Science. The search covered all articles published from database inception to July 30, 2025. Search terms included both medical subject headings (MeSH) and free-text terms: (“Gestational Hypertension” or “Induced Hypertension, Pregnancy” or “Transient Hypertension, Pregnancy” or “Hypertension, Pregnancy Induced” or “Pregnancy Transient Hypertension”) and (“Deformity” or “Congenital Defect” or “Birth Defect” or “Fetal Malformations”) and (“cleft lip- palate” or “oral cleft”). Reference lists and citations were also reviewed to identify additional studies.

### Eligibility criteria

#### Types of studies

Observational studies, including cohort, case-control, and cross-sectional designs.

#### Types of participants

Women with maternal hypertension (pre-pregnancy, gestational, preeclampsia, or chronic hypertension). Pre-pregnancy, chronic hypertension complicating pregnancy is defined as elevated blood pressure that is present before pregnancy (prior to 20 weeks of gestation) or is first diagnosed after 20 weeks of gestation but persists for more than 12 weeks postpartum. Gestational hypertension is defined as hypertension (systolic blood pressure ≥ 140 mmHg and/or diastolic blood pressure ≥ 90 mmHg) that first occurs after 20 weeks of gestation, is not accompanied by significant proteinuria (does not meet the diagnostic criteria for preeclampsia), and typically resolves within 12 weeks postpartum. Preeclampsia is defined as new-onset hypertension (using the same diagnostic thresholds as above) after 20 weeks of gestation, accompanied by at least one of the following: ① Proteinuria ≥ 300 mg per 24-hour urine collection, or a urinary protein/creatinine ratio ≥ 0.3. ② End-organ dysfunction.

#### Observation results

Hypertensive disorder status and the incidence of CLP in offspring. CLP diagnosis and classification were confirmed by trained pediatricians through standard clinical examinations. Syndromic CLP cases were excluded.

#### Exclusion criteria

(a) Reviews, case reports, animal studies, and conference abstracts.
(b) Studies with overlapping data (duplicates excluded).
(c) Studies without extractable effect estimates or outcome data.
(d) Studies focusing primarily on antihypertensive medication exposure (excluded from primary analyses to isolate the effect of the hypertensive state itself; medication-related risks were addressed separately as a secondary objective).

### Data extraction

Screening of titles and abstracts, eligibility assessment, data extraction, and risk-of-bias evaluation were conducted independently by two investigators (Cui and Zhou). Adjusted effect estimates from the primary studies, when available, were preferentially used in our synthesis. Discrepancies were resolved through discussion, and if unresolved, a third author (Song) made the final decision. Extracted information included the first author's surname, publication year, study design, country, study quality, sample size, adjusted confounding factors (if any), and adjusted estimates. We extracted the full set of covariates included in the most-adjusted model from each study and categorized adjustment into prespecified domains: maternal demographics (age), metabolic factors (diabetes, body mass index), behavioral factors (smoking), reproductive/obstetric factors (parity, placental complications), and familial/genetic susceptibility (family history of CLP). A structured adjustment summary is provided in Supplementary Table S1.

### Quality assessment

The Newcastle-Ottawa Scale was used to assess the methodological quality of included cohort and case-control studies (22), with a maximum score of 9. We classified the study quality into three categories: a high quality score of 7 to 9 points, scores from 4 to 6 representing moderate quality, and scores from 0 to 3 indicating low quality. A cross-sectional study was conducted using the Assessment of Healthcare Quality and Research Institutions in the United States, with a total score of 11 points categorized into three grades: scores ranging from 8 to 11 indicated high quality, scores from 4 to 7 represented medium quality, and scores from 0 to 3 corresponded to low quality.

### Statistical analysis and risk of bias assessment

Data analysis was performed using Review Manager 5.3 and Stata 18.0. Effect sizes were reported as odds ratios (ORs) with 95% confidence intervals (CIs). We adopted the random-effects model for data analysis. Statistical significance was defined as a *p*-value <0.05. Heterogeneity was assessed using the I^2^ statistics (significant if I^2^ > 50%) and the Q test (significant if *P* < 0.10) (23). Initially, we employed a fixed effects model. If significant heterogeneity was identified (I^2^ > 50%), the potential causes were then examined. We applied a random-effects model when appropriate. Furthermore, we performed a sensitivity analysis by sequentially excluding one study at a time to assess the robustness of our findings. We have incorporated the results of the random effects model for the primary analysis. The Newcastle-Ottawa Scale was used to assess the risk of bias. The Begg's test was used to visually evaluate the possibility of publication bias (*P* < 0.05) (24). The impact of publication bias on meta-analysis was assessed using the Duval & Tweedie trim-and-fill method. This approach examines how the magnitude of the corrected combined effect changes by estimating and “filling” studies that may be missing. It is used to identify and correct publication bias.

To mitigate confounding inherent to observational studies, we prioritized multivariable-adjusted effect estimates over crude estimates whenever available. Adjusted models most frequently accounted for maternal age, diabetes mellitus, smoking status, body mass index, parity, placental hemorrhage, and family history of cleft lip and palate—factors closely related to both hypertensive disorders of pregnancy and craniofacial anomalies. Prespecified subgroup analyses were conducted according to adjustment status for major maternal confounders to evaluate the stability of effect estimates. In addition, study quality–based subgroup analyses and meta-regression were performed to assess whether residual confounding or methodological characteristics explained between-study heterogeneity. These analytical approaches were implemented to strengthen causal inference while acknowledging the inherent limitations of non-randomized data.

To meaningfully evaluate residual confounding, rather than merely acknowledge it, we implemented a hierarchical analytical strategy. First, we preferentially synthesized multivariable-adjusted estimates controlling for maternal factors known to be strongly associated with both hypertensive disorders and cleft lip and palate, including maternal age, diabetes mellitus, smoking, body mass index, and placental complications. Second, we conducted prespecified subgroup analyses stratified by adjustment status for these factors and directly compared pooled estimates derived from adjusted vs. unadjusted models. Third, meta-regression analyses were used to assess whether study-level differences in adjustment patterns, sample size, or publication period explained between-study heterogeneity. These complementary approaches were employed to evaluate whether observed associations were robust to confounding control, while recognizing the inherent limitations of non-randomized data.

The primary meta-analysis preferentially pooled the most-adjusted estimates available; pooling of crude or minimally adjusted estimates was conducted only as a sensitivity analysis to assess susceptibility to confounding.

## Results

### Literature screening process

**Figure 1:**
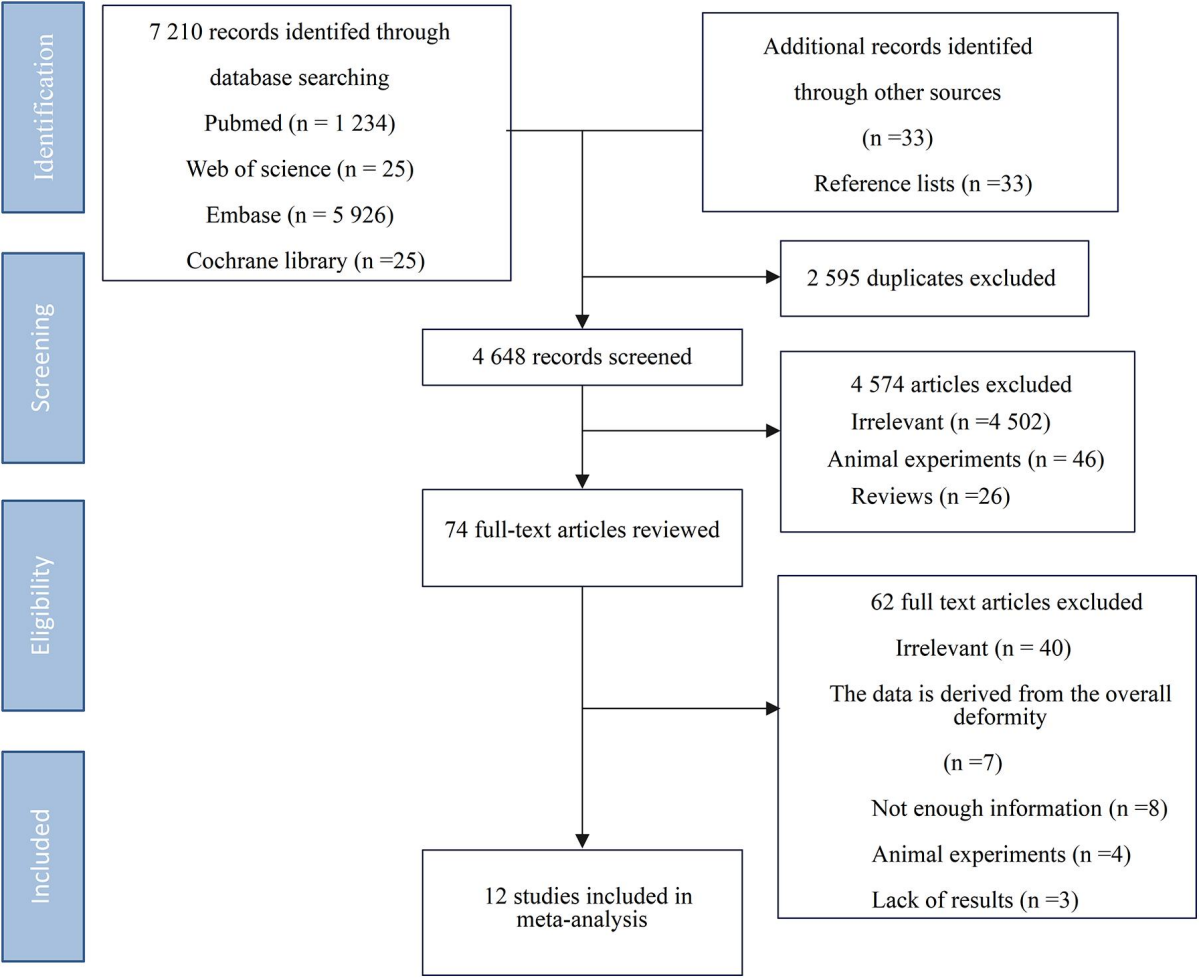
Flowchart of study inclusion and exclusion process.

The initial search identified 7,243 references. After removing 2,595 duplicates, 4,648 titles and abstracts were screened. Based on inclusion/exclusion criteria, 4,574 articles were excluded. Of the 74 full-text articles reviewed, 12 (5–16) studies were ultimately included (Figure 1).

### Characteristics of the included literature

Table 1 presents the basic characteristics of the 12 (5–16) included studies, which consisted of four cohort studies (5–7, 12), seven case-control studies (8, 10, 11, 13–16), and one cross-sectional study (9). In terms of methodological quality, seven studies (5, 6, 8, 10, 12, 14, 15) were rated as high quality and five (7, 9, 11, 13, 16) as moderate quality.

**Table 1:** Characteristics of the included studies.

Author, year	Location	Design	Participants	Cleft lip and palate cases	Age at pregnancy years (y)	Maternal race and ethnicity	Age range for children (d)	Family history of CLP	Effect estimates	Adjusted confounding factors	NOS OR AHRQ
Barbara Luke et al. (2025) (7)	America	Cohort study	1,126,058	138	18–40	Hispanic	Neonate	NA	PLH: ORs = 1.12 (0.76–1.64) PIH: ORs = 1.19 (0.96–1.48)	Paternal age at delivery, race, education, parity, previous caesarean delivery, BMI	5
Hang An et al. (2022) (5)	China	Cohort study	200,215	330	24.90 ± 3.35	White (non-Hispanic), Asian	Neonate	NA	PIH: ORs = 1.3 (0.81–12.08) PE: ORs = 2.54 (1.42–4.57)	Maternal age, BMI, folic acid use, parity, ethnicity, education, occupation, NSOFC	8
Weber et al. (2018) (6)	America	Cohort study	2,499,536	1,859	13–55	NA	Neonate	Yes	HTN: ORs = 1.37 (1.15–1.64)	Maternal age, education, BMI, parity, race/ethnicity, infant sex	7
Anthony H et al. (2018) (8)	America	Case-control study	284	NA	24–35	Asian	Neonate	NA	PIH: ORs = 1.34 (0.44–4.05)	Family history of clefting, family history of cancer, pregnancy-related illnesses and infections, trauma, childbirth complications, immunization history, birth order, ethnic group, smoking, alcohol	9
S. Bellizzi et al. (2016) (9)	Rome	Cross sectional study	310,401	212	20–35	Arab descent, Asian	Neonate	NA	PE: ORs = 8.20 (2.0–34.3) HBP: ORs = 4.20 (2.5–11.6)	Maternal age, status, education, number of deliveries	6
Kishimba et al. (2015) (10)	Tanzania	Case-control study	400	NA	20–40	Caucasians	Neonate	Yes	PIH: ORs = 3.99 (1.67–9.54)	Maternal fever, consanguineous marriage, HIV/AIDS, syphilis, use of alcohol, antiepileptic, multivitamin, low birth weight newborn, paternal age	8
Figueiredo et al. (2015) (11)	America	Case-control study	750	NA	NA	White (non-Hispanic), Black (non-Hispanic), Asian	Neonate	NA	PLH: ORs = 2.6 (1.3–5.1)	Mother and father's status, education level, age, location at birth, family history, alcohol, smoking, household tobacco, chemical use, child's sex	6
Bateman et al. (2014) (12)	America	Cohort study	87,126	NA	20–39	White (non-Hispanic)	Neonate	NA	HBP: ORs = 1.30 (0.6–2.6)	Maternal age, race/ethnicity, region of delivery, year of delivery, obesity, tobacco use, alcohol abuse, illicit drug use/abuse	7
Ferenc Banhidy et al. (2014) (13)	Hungary	Case-control study	60,994	40	19–40	White (non-Hispanic)	Neonate	NA	PE: ORs = 1.1 (0.7–1.7)	Maternal age, birth order, categorical, employment status, pregnancy supplements	5
Ferenc Banhidy et al. (2011) (14)	Hungary	Case-control study	60,994	50	19–40	Indians	Neonate	NA	HBP: ORs = 1.1 (0.8–1.6)	Maternal age, birth order, employment status, acid use, anti-hypertensive drug treatments, folic	7
Wyszynski et al. (2002) (15)	America	Case-control study	7,308	152	18–40	NA	Neonate	NA	HNT: ORs = 1.7 (1.0–2.9) PIH: ORs = 1.3 (1.0–1.6) PE: ORs = 1.9 (0.9–3.8)	Gestational age, maternal age, educational attainment, tobacco consumption, race, status, residence, previous live births	8
Silva et al. (2024) (16)	Brazil	Case-control study	53,188	190	NA	White (non-Hispanic)	Neonate	NA	HTN: ORs = 1.35 (1.239–1.484)	Maternal age, ethnicity, birth order, history of abortion, interval since the last pregnancy, number of prenatal care visits, month of prenatal care began, cigarettes smoked before or during pregnancy, previous preterm birth, infertility treatment, previous cesarean delivery, infections during pregnancy, plurality, child's sex	5

AHRQ, Agency for Health Research and Quality; AIDS, acquired immunodeficiency syndrome; BMI, Body Mass Index; HBP, high blood pressure; HIV, human immunodeficiency virus; HTN, hypertension; NSOFC, nonsyndromic orofacial clefts; PE, pre-eclampsia; PIH, pregnancy-induced hypertension; PLH, pre-gestational hypertension; NA, not applicable; NOS, Newcastle-Ottawa scale; OR, odds ratio.

### Meta-analysis of maternal hypertension and cleft lip and palate

**Figure 2:**
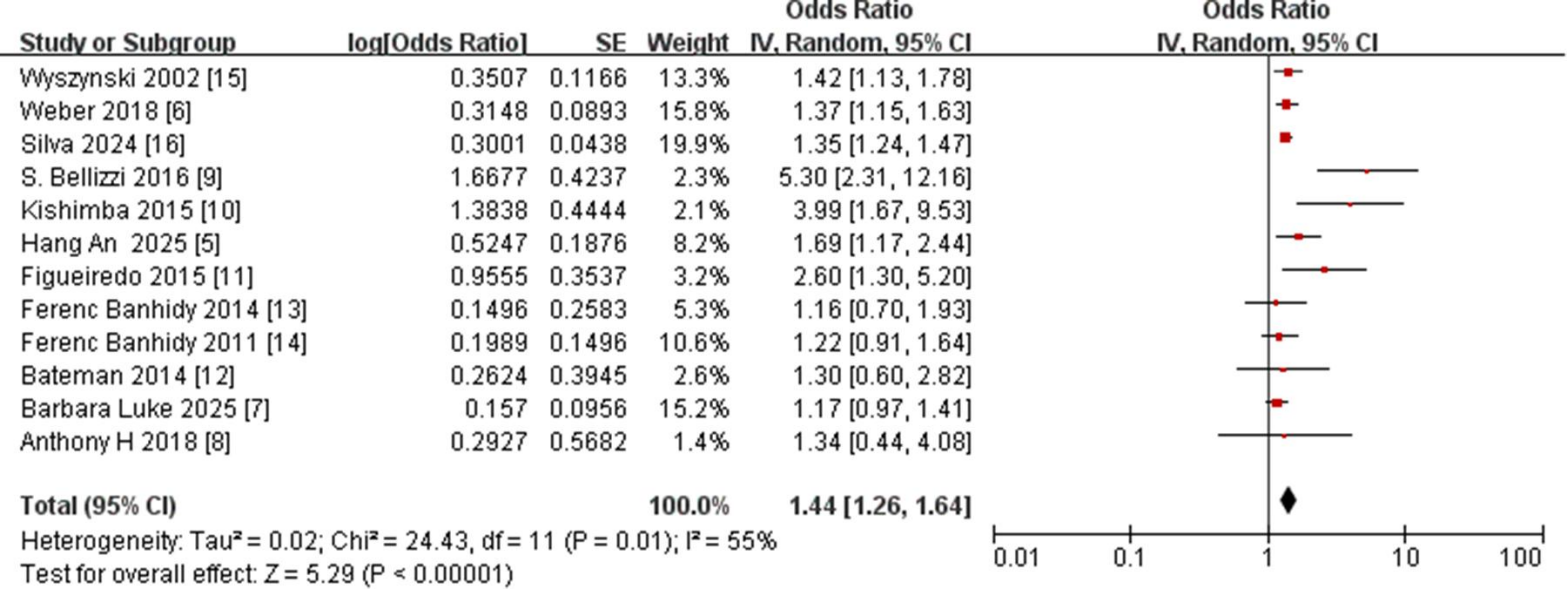
Forest plot of maternal hypertension and CLP in offspring. OR, odds ratio; CI, confidence interval. The total sample size was 4,407,254 with maternal hypertension during the entire period.

The 12 (5–16) included studies involved 4,407,254 participants. Meta-analysis revealed an association between maternal hypertension and CLP risk in offspring (OR = 1.44, 95% CI = 1.26, 1.64, *p* < 0.001). Significant heterogeneity was observed among these studies (I^2^ = 55%; *P* = 0.01) (Table 2 and Figure 2). According to the sensitivity analysis, after excluding any one study, the overall aggregated results did not change significantly (Table 3).

**Table 2:** Results of meta-analysis, test of publication bias and trim and fill analysis.

Category	No. of studies	Effect size	Heterogeneity		Publication bias		Trim-and-fill method	
		OR (95% CI)	*I^2^*, %	*P*	Begg's test (*z, P*)	Egger's test (*t, P*)	No. of potential missing studies	Filled estimates
Maternal hypertension and cleft lip and palate	12	1.44 (1.26–1.64)	55	0.01	1.17, 0.244	1.96, 0.079	0	1.45 (1.27–1.67)
Maternal PEH and cleft lip and palate	2	1.63 (0.72–3.70)	78	0.03	–	–	–	–
Maternal PLH and cleft lip and palate	5	1.36 (1.06–1.74）	43	0.14	–	–	–	–
Maternal PE and cleft lip and palate	4	2.10 (1.11–3.96)	70	0.02	–	–	–	
Maternal hypertension with cleft lip and palate								
Adjusted results	8	1.38 (1.27–1.48)	0	0.436	–	–	–	–
Unadjusted results	4	1.19 (1.01–1.38)	0	0.994	–	–	–	–

PE, pre-eclampsia; PLH, pregnancy-induced hypertension; PEH, preexisting hypertension.

**Table 3:** Sensitivity analysis.

Study excluded	PLH			PIH			PE			Summary results		
	OR (95% CI)	*I^2^*(%)	*P*	OR (95% CI)	*I^2^*(%)	*P*	OR (95% CI)	*I^2^*(%)	*P*	OR (95% CI)	*I^2^*(%)	*P*
Maternal hypertension and cleft lip and palate in offspring												
Barbara Luke et al. (2025) (7)	2.60 (1.33–5.10)	–	0.005							1.66 (1.68–2.61)	61	0.006
Figueiredo et al. (2017) (11)	1.12 (0.76–1.65)	–	0.57							1.49 (1.18–1.88)	56	0.01
Barbara Luke et al. (2025) (7)				1.54 (1.04–2.30)	50	0.11				1.69 (1.28–2.23)	59	<0.01
Anthony H et al. (2018) (8)				1.38 (1.05–1.82)	57	0.07				1.59 (1.24–2.03)	63	<0.01
Kishimba et al. (2015) (10)				1.24 (1.06–1.45)	0	0.96				1.46 (1.18–1.81)	51	0.03
Wyszynski et al. (2002) (15)				1.50 (0.98–2.29）	57	0.07				1.67 (1.25–2.22)	63	<0.01
Hang An et al. (2022) (5)				1.42 (1.03–1.95)	57	0.07				1.62 (1.25–2.11)	63	<0.01
S. Bellizzi et al. (2016) (9)							1.69 (0.99–2.89)	62	0.07	1.59 (1.324–2.03)	56	0.02
Ferenc Banhidy et al. (2014) (13)							2.68 (1.49–4.83)	38	0.20	1.74 (1.30–2.32)	66	<0.01
Wyszynski et al. (2002) (15)							2.32 (0.94–5.76)	80	0.007	1.61 (1.22–2.13)	66	<0.01
Hang An et al. (2022) (5)							2.04 (0.86–4.87)	74	0.02	1.54 (1.31–1.82) 1.52 (1.18–1.97)	59	0.01

PE, pre-eclampsia; PIH, pregnancy-induced hypertension; PLH, pre-gestational hypertension.

**Figure 3:**
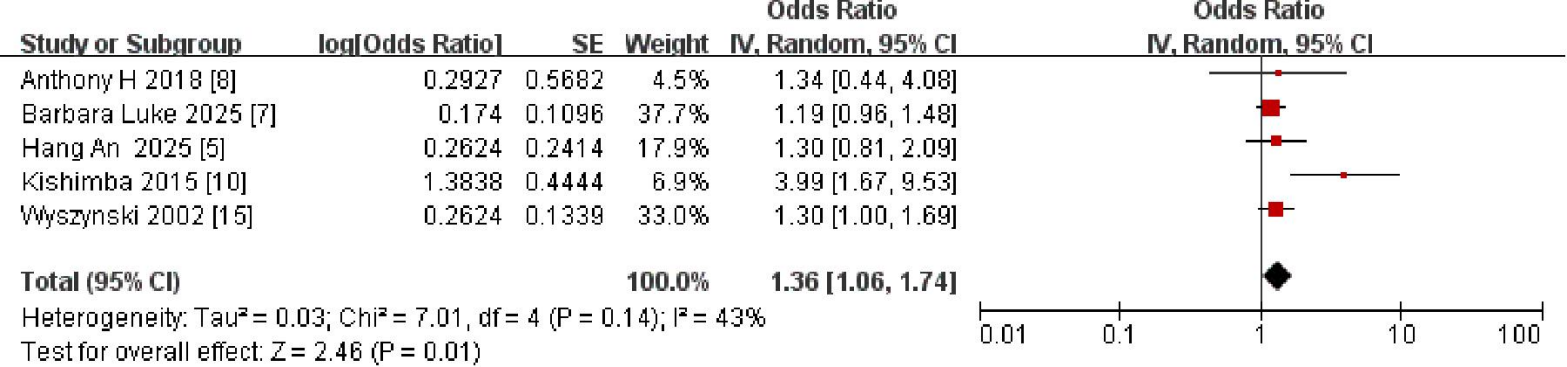
Forest plot of the association between maternal gestational hypertension and CLP in offspring. OR, odds ratio; CI, confidence interval. The total sample size was 1,334,265 with gestational hypertension during pregnancy.

Among the 12 (516) articles we analyzed, a meta-analysis of 5 (5, 7, 8, 10, 15) studies estimated an association between maternal gestational hypertension and CLP in offspring (OR: 1.36, 95% CI: 1.06–1.74). There was no statistical evidence of significant heterogeneity among the studies (I^2^ = 43%, *P* = 0.01) (Figure 3).

**Figure 4:**
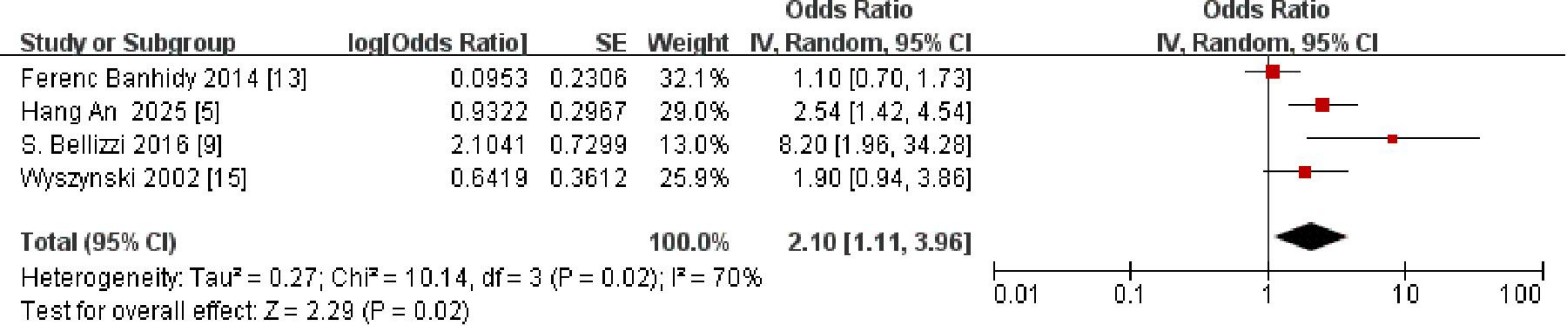
Forest plot of the association between maternal preeclampsia and cleft lip and palate in offspring. OR, odds ratio; CI, confidence interval. The total sample size was 578,918 with preeclampsia during pregnancy.

Regarding preeclampsia, a meta-analysis of four (5, 9, 13, 15) studies found an association between maternal preeclampsia and CLP in offspring (OR: 2.10, 95% CI: 1.11–3.96) (Figure 4). High heterogeneity was detected (I^2^ = 70%; *P* = 0.02). Excluding the study by Ferenc Bánhidy et al. (2012) reduced heterogeneity markedly (from 70% to 38%). There was no alteration in either the direction or the statistical significance of the effect estimate, suggesting the robustness of the finding. Overall results were not significantly influenced by any individual study.

**Figure 5:**
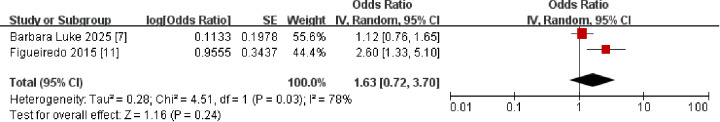
Forest plot of the association between maternal pre-pregnancy hypertension and cleft lip and palate in offspring. OR, odds ratio; CI, confidence interval. The total sample size was 1,126,808 with pre-pregnancy hypertension.

Two (7, 11) studies on maternal pre-pregnancy hypertension indicated no association between maternal pre-pregnancy hypertension and CLP risk in offspring (OR: 1.63, 95% CI: 0.72–3.70) (Figure 5).

Table 4 shows the detailed outcomes of the subgroup analysis. The pooled OR for four cohort studies (5–7, 12) was 1.29 (95% CI: 1.13–1.45). The pooled OR for seven case-control studies (8, 10, 11, 13–16) was also 1.35 (95% CI: 1.24–1.46). Seven high-quality studies (5, 6, 8, 10, 12, 14, 15) yielded a combined OR of 1.38 (95% CI: 1.21–1.54), whereas five moderate-quality studies (7, 9, 11, 13, 16) produced the same result 1.25 (95% CI: 1.08–1.42). Three studies (7, 12, 16) adjusting for maternal diabetes had a combined OR of 1.14 (95% CI: 1.09–1.19), whereas nine (5, 6, 8–11, 13–15) unadjusted studies produced a similar OR of 1.39 (95% CI: 1.23–1.56). Ten studies (6–15) adjusted for maternal age had a combined OR of 1.24 (95% CI: 1.03–1.46), whereas two studies (5, 16) that were unadjusted reported the same OR of 1.37 (95% CI: 1.21–1.53). Two studies (8, 11) adjusted for family history of CLP had a combined OR of 4.73 (95% CI: 2.71–6.76), whereas ten (5–7, 9, 10, 12–16) studies that were unadjusted resulted in an OR of 1.33 (95% CI: 1.24–1.42). Two s**t**udies (8, 11) that adjusted for maternal smoking had a combined OR of 1.33 (95% CI: 1.24–1.42), whereas ten (5–7, 9, 10, 12–16) studies that were unadjusted had the same OR of 1.94 (95% CI: 0.63–3.25). Two studies (7, 15) that adjusted for placental hemorrhage had a combined OR of 1.64 (95% CI: 1.16–2.12), whereas ten (5, 6, 8–14, 16) studies were unadjusted and had the same OR of 1.35 (95% CI: 1.25–1.45).

**Table 4:** Subgroup analysis of the association between maternal hypertension and CLP in offspring.

Variables	Adjusted results					Unadjusted results				
	No. of studies	Effect size OR (95%CI)	Heterogeneity		*P* for the subgroup	No. of studies	Effect size OR (95%CI)	Heterogeneity		*P* for the subgroup
			*I ^2^, %*	*P*				*I ^2^, %*	*P*	
Study design					0.086					0.376
Cohort	3	1.41 (1.18–1.63)	–	–		1	1.17 (0.97–1.42)	0	0.639	
Case-control	4	1.37 (1.25–1.48)	14.3	0.321		3	1.22 (0.87–1.58)	0	0.991	
Cross-sectional study	1	5.30 (2.31–12.17)	–	–		–	–	–	–	
Study quality					0.594					0.673
Moderate	3	1.36 (1.24–1.48)	51	0.128		2	1.17 (0.95–1.39)	0	0.151	
High	5	1.42 (1.23–1.60)	0	0.636		2	1.22 (0.87–1.58)	0	0.898	
Sample size					0.530					–
<10,000		1.47 (1.15–1.78)	1.5	0.385		–	–	–	–	
＞10,000	8	1.42 (1.23–1.60)	0	0.516		–	–	–	–	
Adjusted for maternal diabetes					0.004					–
Yes	3	1.14 (1.09–1.19)	99	<0.001		–	–	–	–	
No	9	1.39 (1.23–1.56)	0	0.419		–	–	–	–	
Adjusted for maternal age					0.231					–
Yes	10	1.24 (1.03–1.46)	88	<0.001		–	–	–	–	
No	2	1.37 (1.21–1.53)	6	0.303		–	–	–	–	
Adjust the family history of cleft lip and palate					0.001					–
Yes	2	4.73 (2.71–6.76)	0	0.908		–	–	–	–	
No	10	1.33 (1.24–1.42)	0	0.505		–	–	–	–	
Adjusted for maternal smoking					0.363					–
Yes	2	1.33 (1.24–1.42)	0	0.505		–	–	–	–	
No	10	1.94 (0.63–3.25)	64 0	0.001 0.346		–	–	–	–	
Adjusted for maternal BMI					0.332					–
Yes	5	1.11 (1.05–1.17)	65	0.021		–	–	–	–	
No	7	1.19 (1.04–1.34)	5	0.389		–	–	–	–	
Adjusted for maternal abortion					0.689					–
Yes	2	1.41 (1.03–1.79)	0	0.443		–	–	–	–	
No	10	1.33 (1.23–1.42)	7	0.375		–	–	–	–	
Adjusted for placental hemorrhage					0.246					–
Yes	2	1.64 (1.16–2.12)	0	0.505		–	–	–	–	
No	10	1.35 (1.25–1.45)	0	0.489		–	–	–	–	

BMI, body mass index; OR, odds ratio.

Unadjusted effect sizes indicate a crude exposure-outcome association that may be confounded by factors such as age or comorbidities. In contrast, adjusted effect sizes statistically account for these known confounders, thereby providing a more valid approximation of the true relationship. Consequently, we prioritized adjusted estimates in our meta-analysis (including subgroup analysis, meta-regression analysis), as they provide more reliable evidence with lower potential for bias.

Notably, adjustment for major maternal confounders resulted in modest attenuation of effect estimates but did not eliminate the association between maternal hypertension and cleft lip and palate. This pattern suggests partial confounding by correlated maternal risk factors, while indicating that hypertension is unlikely to function solely as a proxy exposure.

### Sensitivity analysis

We conducted a sensitivity analysis by sequentially omitting each study. The results consistently showed stable outcome estimates, demonstrating the robustness of the pooled ORs. These findings confirmed the stability of the meta-analysis and reinforced the association between maternal hypertension and cleft lip and palate risk in offspring.

### Risk of bias assessment

**Figure 6:**
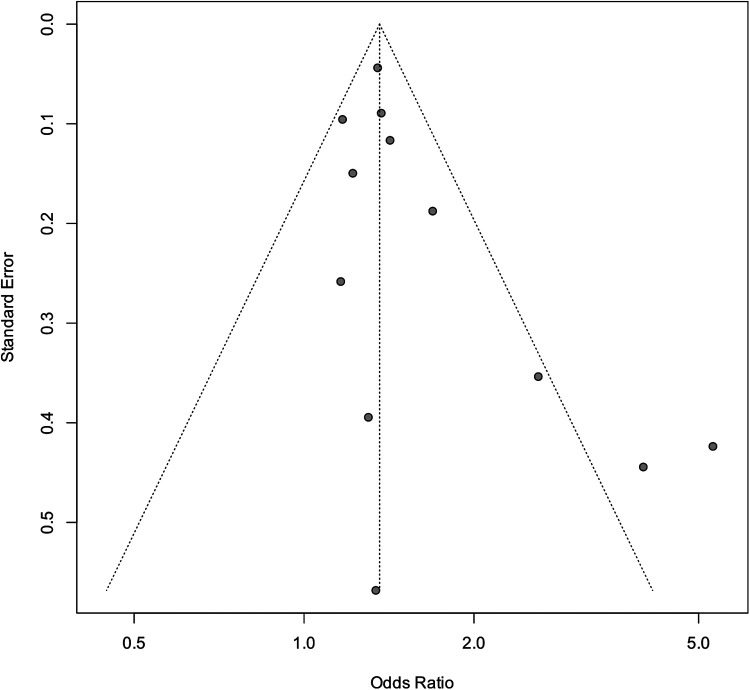
Funnel plot of maternal hypertension and CLP in offspring. OR, odds ratio; SE, standard error. The total sample size was 4,407,254 with maternal hypertension during the entire period.

 Table 2 presents the results of the publication bias and trim-and-fill analyses. The funnel plot suggested potential publication bias (Figure 6). Duval & Tweedie trim-and-fill method was applied to estimate the number of potentially missing studies that may have contributed to the observed asymmetry. The analysis showed no missing studies, indicating that the original results were stable [total: OR 1.44 (95% CI: 1.26–1.64); after trimming and filling: 1.45 (95% CI: 1.27–1.67)].

### Subgroup and meta-regression analyses

Subgroup analyses demonstrated that the association between maternal hypertension and cleft lip and palate persisted in studies adjusting for major maternal confounders, although effect sizes were modestly attenuated. Meta-regression identified sample size and publication year as significant contributors to heterogeneity, whereas study design and geographic region did not materially modify the pooled estimates.

To quantify the likely range of effects across future settings, we additionally calculated the random-effects 95% prediction interval, which reflects between-study heterogeneity and provides a direct measure of generalizability beyond the pooled estimate.

### Heterogeneity exploration

Residual heterogeneity is common in observational perinatal meta-analyses and likely reflects combined influences of (i) varying diagnostic criteria and severity thresholds for hypertensive disorders over time and across health systems, (ii) differences in exposure timing capture relative to craniofacial developmental windows, (iii) variation in outcome ascertainment (registry-based vs. clinically confirmed CLP), and (iv) non-uniform adjustment for confounding domains. Importantly, subgroup and sensitivity analyses indicated that heterogeneity primarily influenced effect magnitude rather than effect direction, supporting the robustness of the association while warranting cautious generalization of the pooled effect size.

## Discussion

### Summary of main findings

In our meta-analysis of 12 (5–16) observational studies involving 4,407,254 participants, we systematically assessed the association between maternal hypertension and cleft lip and palate in offspring. Our analysis indicates that maternal hypertension, including gestational hypertension and preeclampsia, is positively correlated with the risk of CLP.

### Underlying mechanisms linking maternal hypertension and cleft lip and palate

The biological mechanisms discussed below should be interpreted as hypothesis-generating rather than confirmatory, as this meta-analysis synthesizes observational epidemiological data and does not directly assess molecular, experimental, or mechanistic endpoints. Several biological mechanisms, informed by prior *in vitro* and animal studies, could potentially account for the epidemiological link observed in our study. First, we hypothesize that maternal hypertension may lead to placental hypoxia and oxidative stress, based on evidence from previous studies. This hypoxic environment could potentially alter DNA methylation patterns of the Pax3 gene, thereby affecting the fate determination of neural crest cells. This may lead to the failure of craniofacial fusion (25–27). Previous studies showed that women with preeclampsia have signs of endotheliosis, such as cystatin C expression, which might indicate the presence of a metabolic disturbance in an early stage (28, 29). In addition, analyses of the molecular mechanisms of craniofacial development revealed the involvement of several signaling pathways, including the BMP, FGF, Shh and Wnt-signaling pathways, which play crucial roles in midfacial morphogenesis and lip/palate fusion (30, 31). Metabolic disturbance may affect the formation of these pathways. Direct measurement of relevant molecular indicators was not performed in this study. Further studies are needed to investigate their relationships. These proposed mechanisms are largely extrapolated from experimental and animal studies and cannot be directly evaluated using the data included in this review. Accordingly, they should not be interpreted as evidence of causality.

Given this review synthesizes observational epidemiologic studies and includes no molecular, placental, or experimental measurements, mechanistic interpretation should be regarded as biological plausibility rather than evidence of causality. At a high level, hypertensive disorders of pregnancy are characterized by placental vascular dysfunction and impaired uteroplacental perfusion, which can increase the likelihood of fetal hypoxia, oxidative stress, and systemic inflammation. Early craniofacial morphogenesis is sensitive to disturbances in oxygen delivery, nutrient transport, and the intrauterine redox environment; therefore, a vascular–placental pathway provides a coherent explanation for how hypertensive pregnancy contexts could be linked to CLP risk. Importantly, the purpose of this mechanistic framing is to contextualize the observed association—not to infer specific molecular pathways— given that such pathways were not directly assessed in the included studies.

**Figure 7:**
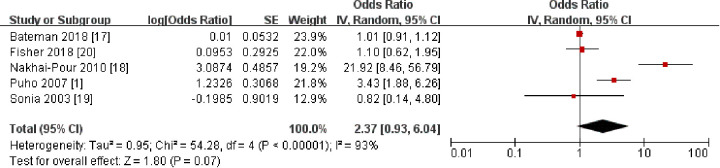
Forest plot of the association between maternal antihypertensive medications and cleft lip and palate in offspring. OR, odds ratio; CI, confidence interval. The total sample size was 606,025 with antihypertensive medications during pre-pregnancy and pregnancy.

**Figure 8:**
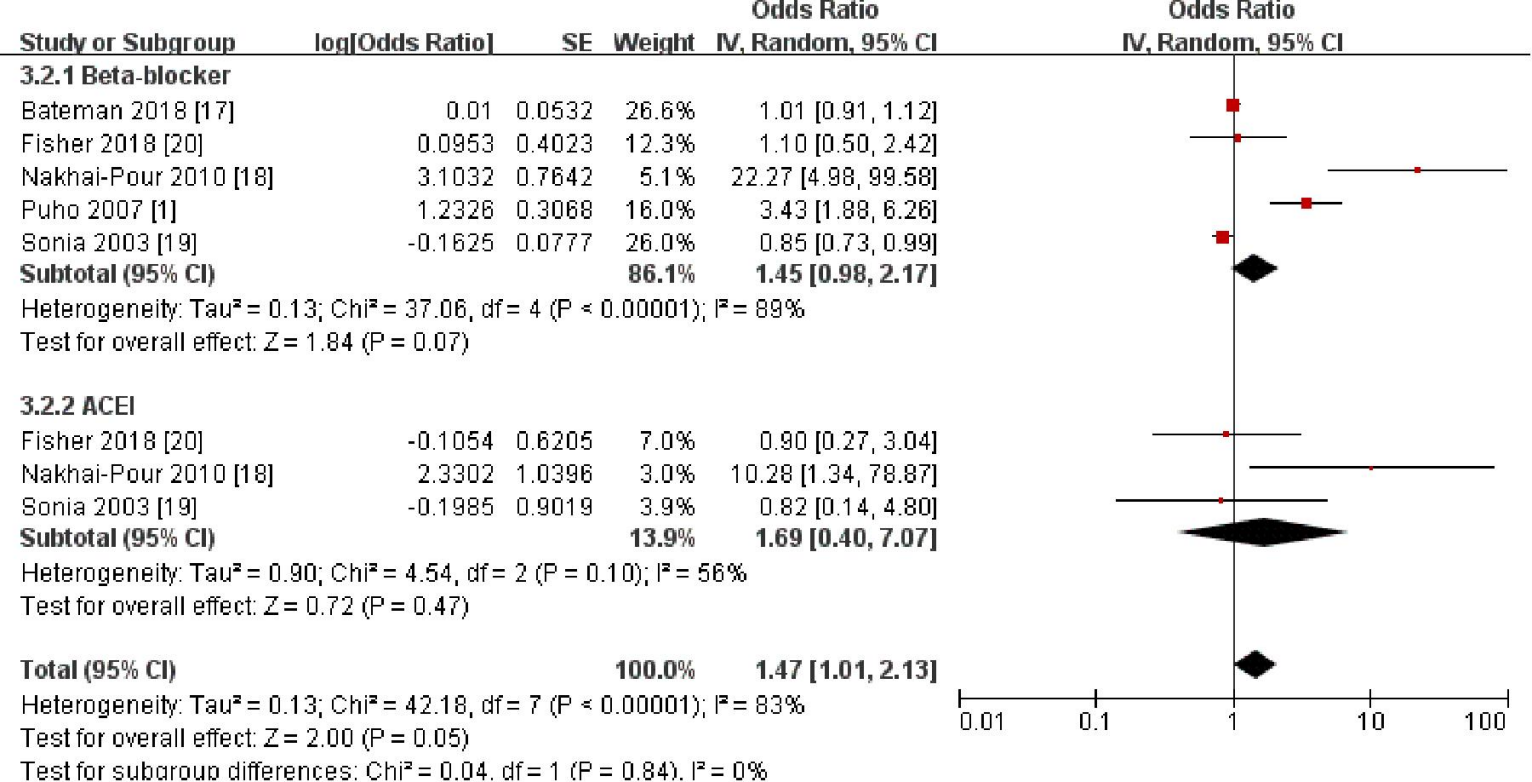
Forest plot of the association between different types of antihypertensive drugs and cleft lip and palate in offspring. OR, odds ratio; CI, confidence interval. The total sample size was 606,025 with antihypertensive medications during pre-pregnancy and pregnancy.

Mothers with hypertension often take antihypertensive drugs, some of which cross the placental barrier. The relationship between antihypertensive medications and CLP in offspring needs to be considered. We therefore included five studies (1, 17–20) involving 606,025 participants to investigate the relationship between maternal antihypertensive medication use and the incidence of CLP in offspring (Figure 7). Subgroup analyses of specific drug categories (e.g., ACE inhibitors, beta-blockers) (Figure 8) implied that these findings should be interpreted as inconclusive evidence. However, current evidence remains limited, and further research is needed.

Distinguishing effects of the hypertensive disease state from effects of antihypertensive treatment is also challenging because treatment is strongly linked to disease severity (confounding by indication). Although medication-exposure studies were excluded from the primary analysis, many women in the hypertension-exposed groups likely received therapy. Thus, the pooled estimate should be interpreted as reflecting the hypertensive pregnancy context rather than a purely medication-independent disease effect.

Our analysis revealed significant heterogeneity (I^2^> 50%). Therefore, we conducted a series of subgroup analyses to explore potential sources. We stratified data by research design (cohort vs. case-control), sample size, study quality (moderate vs. high), different types of hypertension and confounding factors. We found that heterogeneity was reduced within the case-control subgroup, likely attributable to the inherent methodological differences between study designs. Similarly, heterogeneity was lower in studies with larger sample sizes, suggesting that the robust design of larger studies enables more comprehensive control of confounding variables. Subgroup analysis based on gestational hypertension also showed reduced heterogeneity, which may be explained by distinct pathogenic mechanisms and diagnostic period. Gestational hypertension typically develops after 20 weeks of gestation, when craniofacial development is largely complete, whereas chronic hypertension present from early pregnancy may exert more profound effects on early embryonic development. Additionally, the diagnostic criteria and management strategies differ between these conditions, which may contribute to the observed heterogeneity. Furthermore, heterogeneity was low in subgroups defined by placental hemorrhage, high study quality, and maternal smoking. Placental hemorrhage-induced dysfunction can cause fetal hypoxia and metabolic disturbances, potentially leading to failure of craniofacial fusion. Maternal smoking can reduce maternal blood oxygen levels, which may cause chronic hypoxia in fetal tissues. Facial neural crest cells are extremely sensitive to oxygen, such as the proliferation, migration and differentiation process are susceptible to interference. The subgroup analysis in this study is exploratory, primarily aimed at describing potential patterns of effect differences across subgroups and generating hypotheses for future research. Future studies adjusting for these confounding factors are necessary to further explore these associations.

We conducted meta-regression analyses to examine the potential influence of several factors on the results, including study type, study quality, sample size, publication year, different countries and the definition of hypertension. The meta-regression analysis identified the sample size and publication year as major sources of heterogeneity (Table 6). In addition, we applied the GRADE framework to assess the quality of the included studies (Table 5).

**Table 5:** GRADE classification of the included researches.

Certainty assessment							Effect		Certainty	Importance
No of study	Study design	Risk of bias	Inconsistency	Indirectness	Imprecision	Other considerations	Relative (95% CI)	Absolute (95% CI)		
Barbara Luke et al. (2025) (7)	Non-randomised studies (Cohort study)	Serious (Confounding bias)	Not serious	Not serious	Not serious	None	OR 1.17 (0.97 to 1.42)	1 fewer per 1,000 (from 1 fewer to 1 fewer)	⊕⊕⊕◯ Moderate	5-IMPORTANT
Hang An et al. (2022) (5)	Non-randomised studies (Cohort study)	Not serious	Not serious	Not serious	Not serious	None	OR 1.69 (1.17 to 2.44)	2 fewer per 1,000 (from 2 fewer to 1 fewer)	⊕⊕⊕⊕ High	8-CRITICAL
Weber et al. (2018) (6)	Non-randomised studies (Cohort study)	Serious (Confounding bias)	Not serious	Not serious	Not serious	None	OR 1.37 (1.15 to 1.64)	1 fewer per 1,000 (from 2 fewer to 1 fewer	⊕⊕⊕◯ Moderate	7-CRITICAL
Anthony H et al. (2018) (8)	Non-randomised studies (Case-control study)	Not serious	Not serious	Not serious	Not serious	None	OR 1.34 (0.44 to 4.05)	0 fewer per 1,000 (from 0 fewer to 0 fewer)	⊕⊕⊕⊕ High	9-CRITICAL
S. Bellizzi et al. (2016) (9)	Non-randomised studies (Cross sectional study)	Serious (Confounding bias)	Not serious	Not serious	Not serious	Strong association all plausible residual confounding would reduce the demonstrated effect	OR 5.30 (2.31 to 12.17)	5 fewer per 1,000 (from 12 fewer to 2 fewer)	⊕⊕⊕⊕ High	6-IMPORTANT
Kishimba et al. (2015) (10)	Non-randomised studies (Case-control study)	Not serious	Not serious	Not serious	Not serious	Strong association	OR 3.99 (1.67 to 9.54)	0 fewer per 1,000 (from 0 fewer to 0 fewer)	⊕⊕⊕⊕ High	8-CRITICAL
Figueiredo et al. (2015) (11)	Non-randomised studies (Case-control study)	Serious (Confounding bias)	Not serious	Not serious	Not serious	None	OR 2.6 (1.3 to 5.1)	0 fewer per 1,000 (from 0 fewer to 0 fewer)	⊕⊕⊕◯ Moderate	6-IMPORTANT
Bateman et al. (2014) (12)	Non-randomised studies (Cohort study)	Serious (Confounding bias)	Not serious	Not serious	Not serious	None	OR 1.30 (0.6 to 2.6)	1 fewer per 1,000 (from 3 fewer to 1 fewer)	⊕⊕⊕◯ Moderate	7-CRITICAL
Ferenc Banhidy et al. (2014) (13)	Non-randomised studies (Case-control study)	Serious (Confounding bias)	Not serious	Not serious	Serious (Confounding bias)	None	OR 1.10 (0.7 to 1.7)	0 fewer per 1,000 (from 0 fewer to 0 fewer)	⊕⊕◯◯ Low	5-IMPORTANT
Ferenc Banhidy et al. (2011) (14)	Non-randomised studies (Case-control study)	Serious (Confounding bias)	Not serious	Not serious	Not serious	None	OR 1.10 (0.8 to 1.6)	0 fewer per 1,000 (from 0 fewer to 0 fewer)	⊕⊕⊕◯ Moderate	7-CRITICAL
Wyszynski et al. (2002) (15)	Non-randomised studies (Case-control study)	Serious (Confounding bias)	Not serious	Not serious	Not serious	None	OR 1.42 (1.13 to 1.78)	0 fewer per 1,000 (from 0 fewer to 0 fewer)	⊕⊕⊕◯ Moderate	8-CRITICAL
Silva et al. (2024) (16)	Non-randomised studies (Case-control study)	Serious (Confounding bias)	Not serious	Not serious	Serious (Confounding bias)	None	OR 1.350 (1.239 to 1.484)	0 fewer per 1,000 (from 0 fewer to 0 fewer)	⊕⊕◯◯ Low	5-IMPORTANT

The concern that maternal hypertension may operate as a proxy for closely associated metabolic, behavioral, or sociodemographic factors is well founded and represents a central challenge in observational perinatal research. In the present study, this issue was addressed analytically through comparison of pooled estimates derived from confounder-adjusted and unadjusted models. Although effect sizes were attenuated after adjustment, the direction and statistical significance of the association persisted across strata adjusting for maternal age, diabetes, smoking, body mass index, and placental complications. This pattern is consistent with partial—but not complete—confounding and argues against maternal hypertension being solely a surrogate marker for these related exposures. However, because several relevant determinants, particularly socioeconomic status, nutritional factors, and severity or duration of hypertension, were inconsistently measured or unavailable across studies, residual confounding cannot be fully excluded.

Our analysis shows that maternal hypertension itself is associated with increased CLP risk in offspring. When investigating CLP risk factors, confounding genetic and environmental influences must be considered. For example, Figueiredo et al. (2015) reported that descendants with a family history of CLP have a higher risk. The results remained unchanged in the subgroup analyses, regardless of whether we adjusted for family history of clefts. Maternal diabetes, age, smoking, excessive body mass index, miscarriage and placental bleeding can increase CLP risk in offspring. The increased CLP risk persisted in subgroup analyses, irrespective of adjustment for major potential confounders, such as maternal diabetes, age, smoking, body mass index, miscarriage, placental bleeding, and family history. Significant heterogeneity was observed in the subgroups after adjusting for maternal diabetes, age, and body mass index. This heterogeneity could be attributed to variations (such as whether variables were parameterized as continuous or categorical, and the appropriateness of the chosen cutoff points) in the adequacy of adjustments across different studies. In subgroup of family history of cleft lip and palate, maternal smoking and placental hemorrhage, we found that the small number of included studies, only two, may introduce bias into the research findings. Future high-quality studies are needed to further investigate the relationship between them.

Importantly, subgroup and sensitivity analyses demonstrated that the direction and statistical significance of the association between maternal hypertension and cleft lip and palate were largely consistent across strata, indicating that heterogeneity primarily influenced effect magnitude rather than effect direction. The persistence of the association after sequential exclusion of individual studies further supports the robustness of the findings. Residual heterogeneity likely reflects unmeasured study-level characteristics, including variation in exposure timing relative to craniofacial development, differential adjustment for confounders, and differences in case ascertainment. Accordingly, subgroup and meta-regression analyses were used to explore—rather than fully resolve—heterogeneity. Future prospective studies with harmonized definitions and standardized exposure assessment are required to further clarify sources of variability.

### Strengths

Our study has several strengths. It included 4,407,254 participants, with extensive global and transnational representation. We were able to differentiate between different hypertension groups in these studies. The results remained consistent throughout the sensitivity analysis

### Limitations

However, this study had certain limitations. First, some of the studies were retrospective, which may have led to recall bias. Second, the results may be subject to residual confounding factors owing to unmeasured or poorly measured factors. Third, inconsistencies in hypertension definitions across studies could have contributed to the heterogeneity, inconsistent reporting of exposure timing may be difficult to fully identify and control all possible confounding factors. Finally, only English-language articles were included, which may introduce language bias.

Residual confounding remains possible due to unmeasured or inconsistently adjusted maternal factors, and the observational nature of the evidence precludes causal inference.

A key limitation inherent to observational studies is confounding bias, stemming from the intricate interplay between maternal hypertension and a constellation of metabolic, behavioral, and sociodemographic risk factors—including obesity, diabetes mellitus, cigarette smoking, and socioeconomic status. Despite our deliberate inclusion of studies that attempted to adjust for these confounders, heterogeneity persists in the comprehensiveness and rigor of such adjustments; furthermore, numerous critical confounders—particularly the multifaceted dimensions of socioeconomic status—are frequently unmeasured or underreported. Consequently, the associations observed herein may be obscured by this complex interplay of factors and should not be construed in isolation as direct effects of hypertension. A further methodological constraint is the inability, based on existing observational data, to disentangle the independent impacts of the “maternal hypertensive disease state” from those of “antihypertensive therapeutic interventions” on offspring outcomes. While our primary objective was to evaluate the intrinsic risk of the pathology itself, the majority of mothers in the included studies likely received clinical management. Thus, our effect estimates may inadvertently encapsulate the potential impact of therapeutic regimens. Additionally, some subgroup or meta-regression analyses included only two studies, given the extremely limited number of studies, these results are highly uncertain and should be regarded as preliminary and descriptive. Future research is needed to validate these findings.

Although this meta-analysis suggests an association between maternal hypertension and CLP in offspring, findings should be interpreted cautiously given possible residual confounding. Evidence regarding maternal antihypertensive drug use remains sparse—only five studies (1, 17–20) to date. Further high-quality research that adequately adjust for confounders is required to evaluate whether maternal hypertension and antihypertensive medication use contribute to CLP risk in offspring.

This study is not merely a simple synthesis of existing evidence. To investigate the association between maternal hypertension and CLP in offspring, this study adjusted for potential confounders and performed subgroup analyses and meta-regression analysis to identify sources of heterogeneity (Table 6). In addition, we applied the GRADE framework to assess and grade the quality of evidence, providing a systematic evaluation of the robustness of our findings (Table 5). This study indicates that maternal hypertension increases the risk of CLP in offspring. It is recommended that healthcare providers intensify rigorous screening and management protocols for hypertensive pregnancies. Concurrently, heightened vigilance must be directed toward the progeny of these mothers, thereby furnishing a reference for the prevention and clinical management of CLP in offspring.

**Table 6:** Meta-regression analysis of maternal hypertension and cleft lip and palate in offspring.

Variable	Coef	*P*	95% CI	
Research type	−0.297	0.576	−0.453	1.286
Research quality	0.208	0.301	−0.438	0.854
Sample size	0.360	0.017	−0.370	1.090
Definition of hypertension	0.013	0.985	−3.172	3.537
Research year	0.686	0.042	−0.105	1.476
Different countries	0.324	0.131	−0.115	0.763

Hypertension diagnostic criteria varied across included studies, reflecting both cross-national variation and changes in clinical guidelines over time. For example, the traditional diagnostic criteria for preeclampsia were based on the coexistence of hypertension and proteinuria. However, the 2013 guidelines issued by the American College of Obstetricians and Gynecologists (ACOG) and subsequent guidelines from the International Society for the Study of Hypertension in Pregnancy (ISSHP) expanded the diagnostic framework to allow a diagnosis of preeclampsia in the presence of hypertension accompanied by evidence of maternal end-organ dysfunction (such as thrombocytopenia or elevated liver enzyme levels), even in the absence of significant proteinuria. Although subgroup analyses suggested that the primary findings were robust to these definitional variations, the results should nonetheless be interpreted with appropriate caution.

## Conclusions

This meta-analysis suggests that maternal hypertension—particularly gestational hypertension and preeclampsia—is associated with a higher risk of cleft lip and palate in offspring. However, given the observational nature of the evidence, residual confounding, exposure misclassification, and confounding by indication related to antihypertensive treatment cannot be excluded; therefore, causal inference is not warranted. Future large, well-characterized prospective cohorts employing standardized definitions, precise exposure timing, and advanced causal-inference approaches are required to clarify whether the association is causal and to disentangle disease effects from treatment effects.

## Data Availability

The datasets presented in this study can be found in online repositories. The names of the repository/repositories and accession number(s) can be found in the article/Supplementary Material.
