# Supplementary material for: Cumulative evidence for the association between maternal hypertension and cleft lip and palate in offspring: a systematic review and meta-analysis
Source: Front Oral Health. 2026 Mar 13;7:1725513. doi: 10.3389/froh.2026.1725513 (PMC13021887; doi:10.3389/froh.2026.1725513)
Supplement: Supplementary file 1 [file Table1.docx]

| **Table S1. Summary of confounder-adjusted estimates for the association between maternal hypertension and CLP** | | | | | | | | | | | |
| --- | --- | --- | --- | --- | --- | --- | --- | --- | --- | --- | --- |
| Author, year | Location | Design | Hypertension phenotype assessed | Maternal age | Diabetes mellitus | BMI | Smoking | Family history of CLP | Placental complications | Socioeconomic factors | Most-adjusted model used |
| Barbara Luke et al. (2025) (7) | America | Cohort study | PLH, PIH | Yes | Yes | Yes | No | No | Yes | Yes | No |
| Hang An et al. (2022) (5) | China | Cohort study | PIH, PE | Yes | No | Yes | No | No | No | Yes | Yes |
| Weber et al. (2018) (6) | America | Cohort study | HTN | Yes | No | Yes | No | Yes | No | Yes | Yes |
| Anthony H et al. (2018) (8) | America | Case-control study | PIH | Yes | No | No | No | No | No | No | No |
| S. Bellizzi et al. (2016) (9) | Rome | Cross sectional study | PE, HBP | Yes | No | No | No | No | No | Yes | Yes |
| Kishimba et al (2015) (10) | Tanzania | Case-control study | PIH | Yes | No | No | No | Yes | No | No | Yes |
| Figueiredo et al. (2015) (11) | America | Case-control study | PLH | No | No | No | Yes | NA | No | No | Yes |
| Bateman et al. (2014) (12) | America | Cohort study | HBP | Yes | Yes | No | No | No | No | No | Yes |
| Ferenc Banhidy et al. (2014) (13) | Hungary | Case-control study | PE | Yes | No | No | No | No | No | Yes | No |
| Ferenc Banhidy et al. (2011) (14) | Hungary | Case-control study | HBP | Yes | No | No | No | No | No | Yes | No |
| Wyszynski et al. (2002) (15) | America | Case-control study | HN, PIH, PE | Yes | No | Yes | No | No | Yes | Yes | Yes |
| Silva et al. (2024) (16) | Brazil | Case-control study | HTN | Yes | Yes | Yes | No | No | No | No | Yes |
| BMI, Body Mass Index; HBP, high blood pressure; HTN, hypertension; PE, pre-eclampsia; PIH, pregnancy-induced hypertension; PLH, pre-gestational hypertension. | | | | | | | | | | | |
